# Composition―Nanostructure Steered Performance Predictions in Steel Wires

**DOI:** 10.3390/nano9081119

**Published:** 2019-08-03

**Authors:** Kun V. Tian, Francesca Passaretti, Adelaide Nespoli, Ernesto Placidi, Roberta Condò, Carla Andreani, Silvia Licoccia, Gregory A. Chass, Roberto Senesi, Paola Cozza

**Affiliations:** 1 Department of Chemical Science and Technologies, Università degli Studi di Roma Tor Vergata, 00133 Roma, Italy; 2 Centro NAST, Università degli Studi di Roma Tor Vergata, 00133 Roma, Italy; 3 Materials Science Research Institute, Faculty of Dentistry, Semmelweis University, 1088 Budapest, Hungary; 4 CNR ICMATE, Institute of Condensed Matter Chemistry and Technologies for Energy, 23900 Lecco, Italy; 5 Istituto di Struttura della Materia (ISM-CNR), 00133 Roma, Italy; 6 Department of Physics, Università degli Studi di Roma Tor Vergata, 00133 Roma, Italy; 7 Department of Clinical Sciences and Translational Medicine, Università degli Studi di Roma Tor Vergata, 00133 Roma, Italy; 8 School of Biological and Chemical Sciences, Queen Mary University of London, London E1 4NS, UK; 9 Department of Chemistry, The University of Hong Kong, Hong Kong, China; 10 Department of Chemistry, McMaster University, Hamilton, ON L8S 4L8, Canada

**Keywords:** steel, wire, martensite, work hardening, gamma energy, neutron, alloying, structure, corrosion, surface

## Abstract

Neutron scattering in combination with scanning electron and atomic force microscopy were employed to quantitatively resolve elemental composition, nano- through meso- to metallurgical structures and surface characteristics of two commercial stainless steel orthodontic archwires—G&H and Azdent. The obtained bulk composition confirmed that both samples are made of metastable austenitic stainless steel type AISI 304. The neutron technique’s higher detection sensitivity to alloying elements facilitated the quantitative determination of the composition factor (CF), and the pitting resistance equivalent number (PREN) for predicting austenite stability and pitting-corrosion resistance, respectively. Simultaneous neutron diffraction analyses revealed that both samples contained additional martensite phase due to strain-induced martensite transformation. The unexpectedly high martensite content (46.20 vol%) in G&H was caused by combination of lower austenite stability (CF = 17.37, *p* = .03), excessive cold working and inadequate thermal treatment during material processing. Together, those results assist in revealing alloying recipes and processing history, and relating these with corrosion resistance and mechanical properties. The present methodology has allowed access to unprecedented length-scale (μm to sub-nm) resolution, accessing nano- through meso-scopic properties. It is envisaged that such an approach can be extended to the study and design of other metallic (bio)materials used in medical sciences, dentistry and beyond.

## 1. Introduction

Ideally, medical (arch)wires are designed to support, retain or move bone and/or tooth positions with light and continuous force towards a pre-planned final alignment. The associated force vectors can be optimized to reduce patient discomfort, prevent tissue hyalinisation and undermine resorption [1]. When in service, archwires should behave elastically for a period of weeks to months, dependent on the treatment phase [2]. Although criteria of an ideal archwire are established, no single archwire satisfies all these requirements [2]. It is therefore the duty of the orthodontist to select appropriate wires during various stages of treatment to obtain the desired final tooth alignment [2,3].

Towards guiding product selection and importantly marketing, most laboratory studies on commercial archwires focus on resolving their response to the relevant physical/chemical changes [4], which normally takes the form of mechanical and corrosion resistance tests that supposedly mimic clinical situations [5]. However, great variation in reported dental literature test data exists, even for fundamental properties, such as Young’s modulus of elasticity; disparate values of 126 to 262 GPa have been published for stainless steel [6]. Even when determined with test design of the highest precision and reproducibility, variation still exists, evidencing industrial processing as being the dominant factor [6]. Predictably, industrial details of material processing are normally kept as commercial secrets [7]. Material processing generally comprises two facets: Initial metal ingot selection, which determines intrinsic composition; and mechanical processing, of which size reduction (rolling and drawing), intermediate heat treatment and surface finishing determine metallurgical structure and surface characteristics. Although surface characteristics are routinely investigated and used to predict properties [8,9,10], there lacks information about the fundamental composition as well as metallurgical and phase structures, which directly influence final mechanical properties [11,12,13,14,15,16]. This is especially pertinent for austenitic stainless steel alloy, due to its high susceptibility to work-hardening effect in both metastable and stable types; composition governs its stability [17].

Austenitic stainless steel remains the most popular archwire alloy type since its introduction as a material for use in medicine and dentistry in the 1950s, due to its combination of high resistance to corrosion, high ductility, good formability, reasonable weldability, low coefficient of friction and low cost [7,18]. The ‘composition factor’ (CF) has been introduced to tractably relate composition to stability [17,19]. For austenitic stainless steel, the CF is defined as follows:

(1) CF=18(C%)+13(N%)+0.90(Ni%)+0.27(Cr%)+0.47(Mn%)+0.53(Mo%)+0.97(Cu%),

where % is wt-%. CF in this case serves as an inverse function of the amount of martensite formed from metastable austenite at room temperature at true strain of 0.2.

Work hardening is the strengthening of a metal by plastic deformation [17], but at the cost of reduced ductility and formability. Metastable austenite undergoes strain-induced transformation to martensite, whilst stable austenite manifests in formation of stacking faults [17].

Towards preventing the work hardened material from cracking, thermal treatment is typically done to restore the original ductility [20], albeit standard treatments fail to rectify these performance faults. X-ray diffraction [11,21], and recent neutron diffraction [7] studies of work hardened stainless steel wires have discovered martensitic phase dominating at 82 vol% [21], rising from excessive cold working and inadequate thermal treatment [7]. Although martensite-transformation increases the yield strength, it significantly reduces ductility and thus fatigue resistance (durability), raising the probability for premature performance failure [22].

Composition also influences corrosion resistance. Besides the minimum 13% chromium (Cr) content for passivity [23], carbon content is essential for stabilizing the austenitic phase, yet is kept at a diminutive ≤0.08%, due to sensitization, which may lead to intergranular corrosion [24]. Consequently, nitrogen is added to mitigate this effect. Molybdenum (Mo) is also added to increase resistance to pitting-corrosion. Steel’s resistance to corrosion at localized sites in a reducing atmosphere can be quantified by its pitting resistance equivalent number (PREN), a predictive measure based on its elemental composition [25]:

(2) PREN=Cr%+3.3(Mo%),

where *%* is wt-%.

Hence, the precise determination of elemental composition and the extent of martensitic phase fractioning of stainless steel is essential for characterizing the influence material processing on performance, serving the dual purpose of guiding clinical practice and product quality control via such material forensics. Further, such tractable measure of final properties will enable tractable optimization of wire fabrication, towards improving desired force-delivery by the wires and their enhanced durability [26].

Established superficial probes used in the industry (microscopy-energy dispersive X-ray spectroscopy (EDX) and XRD) cannot access either sample bulk or nano-scale structural details. Encouragingly, neutron scattering provides a non-destructive route to nine decades of structural resolution (0.01 nm to 1 cm), with an even wider temporal resolution (10^−18^ to 1 s) [26]. Such measurements provide precise details of the elemental composition [27,28] and nano-scale through macrostructures [7,29] of the materials, providing a quantitative history of work hardening effects and informative predictions of bulk material properties.

**Figure 1:**
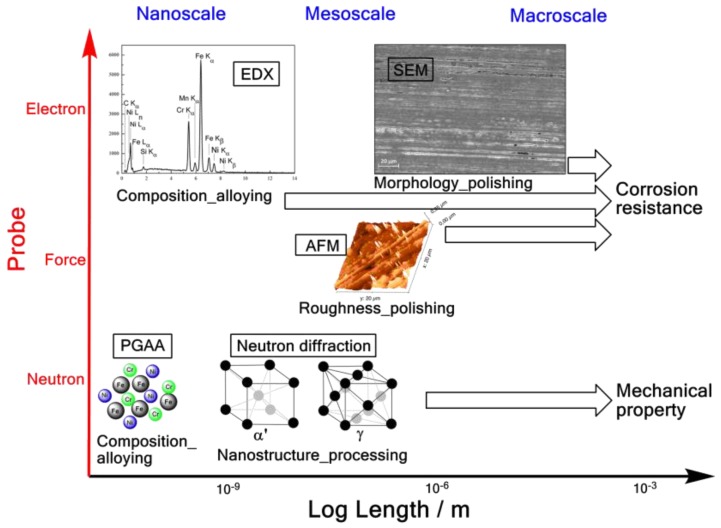
Illustration of the nano- through meso-scale properties and their respective determining factor from the material production process, afforded in this work with different techniques, applying three probes (i.e., neutron, force and electron).

Thus the aim of this work was to investigate the atomistic-through-macroscopic structure of selected functional stainless steel archwires, through a combination of neutron scattering, scanning electron microscopy-energy dispersive X-ray spectroscopy (SEM-EDX) and atomic force microscopy (AFM) (Figure 1), towards resolving composition-structure-property relationship.

## 2. Materials and Methods

### 2.1. Materials

Stainless steel archwires of two commercial brands, one renowned G&H (G&H Wire Company, Franklin, IN, USA) and the previously uncharacterized Azdent (Baistra Industrial, Zhengzhou, China), of size 0.43 × 0.64 mm (0.017 × 0.025 inch) in preformed U-shapes were measured in their as-received form.

### 2.2. Simultaneous Prompt Gamma Activation Alalysis (PGAA) and Neutron Diffraction Measurement

**Figure 2:**
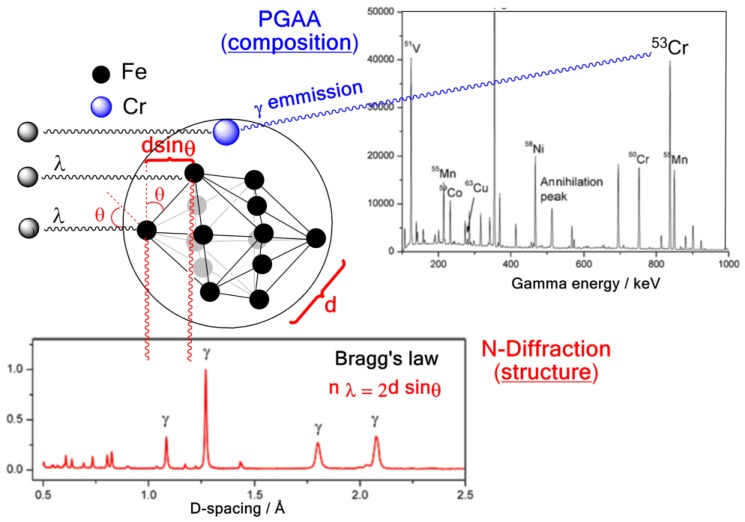
Schematic of simultaneous prompt gamma activation analysis (PGAA) and neutron diffraction measurements carried out. PGAA elemental measurements (**upper**) reveal constituent elements and isotope content, whilst diffraction (**lower**) resolves the d-spacing of the differing γ austenite and α’ martensite phases present and their relative fractional content.

The relevant experimental details of PGAA [7] and those of neutron diffraction [28] have been respectively reported. Herein, with the improved integration capacity afforded on the INES beamline at the ISIS pulsed neutron and muon source (Rutherford Appleton Laboratory, Harwell, UK), coupled with relevant physical, chemical and mechanical material property prediction, we report on the structural and phase-compositional result-trends emerging from integrated and simultaneous PGAA-neutron diffraction measurement (Figure 2).

Initially, a calibrating, empty-instrumental background measurement (no sample) was carried out over an acquisition time of 2400 s in live time. Then the two G&H and Azdent samples, each as a batch of 10 wires, were loaded into the sample holder tanks and measured for 73,435 s (~20.4 h) and 51,722 s (~13.4 h), respectively; with collection times based on acquiring sufficient signal resolution. Care was taken to align the specimen axis perpendicular to the neutron beam direction to maximize measurement efficiency and accuracy.

Neutron diffraction data were analyzed with GSAS (General Structure Analysis System) software [30] for the refinement of structural models to powder diffraction data with the EXPGUI interface [31], using the Rietveld method to determine weight fractions of the main phases. PGAA data were analyzed as such: The instrumental γ background signal was first subtracted from the raw PGAA spectrum to obtain the background-subtracted spectrum for further analysis. The most composition and performance relevant element/isotope γ-ray lines (Fe, Cr, Ni, Mn, Cu, Mo and Co, in this case) were identified (Figure 2) through matching with the major γ-ray energy database [32].

Parallel quantitative standard PGAA measurements of the same two sets of samples were carried out at the Budapest Neutron Center (BNC), Budapest, Hungary to provide benchmark and reference data. Ten 5–10 mm long sections were cut from each batch of samples. They were heat sealed in Teflon bags and loaded into one slot of the automatic sample changer at the NIPS-NORMA station [33,34]. The irradiation and the data collection were carried out using the Budapest NIPS Data Acquisition software. The acquisition time for both samples was equally set to 40,000 s live time; the shorter times relative to INES beamline justified by the higher relevant resolution on the PGAA instrument at BNC. The spectrum evaluation was performed using the HYPERMET PC γ spectrum evaluation software [35].

### 2.3. Simultaneous Elemental-Surface Morphology Measurement with SEM-EDX

Simultaneous elemental-surface morphology measurements were made using a LEO1430 SEM instrument (LEO Electron Microscopy Ltd., Cambridge, UK) with a tungsten filament, equipped with Energy Dispersive Microanalysis EDX (IncaCrystal 300, Oxford Instruments, Abingdon, UK). The main operating parameters used were 20 kV acceleration voltage and 15 mm working distance. An acquisition time of 120 s was applied, with a dead time of 20%. The specimens were degreased with an acetone-bath ultrasonic cleaner before each measurement. Each measurement was performed at three different positions along the full wire length.

### 2.4. Surface Roughness Measurement with AFM

A 5 mm long section was cut from the straight end of one specimen from each G&H and Azdent brand wire, cleaned with ethyl alcohol and dried with nitrogen prior to characterization with a commercial AFM instrument (Veeco Multiprobe Nanoscope IIIa, Veeco, Santa Barbara, CA, USA) operating in contact mode at room temperature. A silicon nitride tip with a 7–10 nm tip curvature radius was mounted on cantilevers with a spring constant of 0.5 N·m^−1^. AFM images were analyzed with the Gwyddion 2.34 Data Processing Software [36]. The surface roughness was determined with AFM morphology, averaging three images of 20 × 20 µm acquired for each specimen.

### 2.5. Statistical Analysis

The overall variance of a determination was taken as the sum of the variances of each component, and those variances were taken as proportions of the individual measurement error in the mean, with multipliers according to the powers involved, following the usual propagation of error rule [37]. The overall proportional error in CF and PREN was therefore determined from the following:

(3) $${\left(\frac{∆CF}{CF}\right)}^{2}={\left(\frac{∆C\%}{C\%}\right)}^{2}+{\left(\frac{∆N\%}{N\%}\right)}^{2}+{\left(\frac{∆Ni\%}{Ni\%}\right)}^{2}+{\left(\frac{∆Cr\%}{Cr\%}\right)}^{2}+{\left(\frac{∆Mn\%}{Mn\%}\right)}^{2}+{\left(\frac{∆Mo\%}{Mo\%}\right)}^{2}+{\left(\frac{∆Cu\%}{Cu\%}\right)}^{2},$$

(4) $${\left(\frac{∆PREN}{PREN}\right)}^{2}={\left(\frac{∆Cr\%}{Cr\%}\right)}^{2}+{\left(\frac{∆Mo\%}{Mo\%}\right)}^{2},$$

The statistical difference between each determined measures for the two samples was tested using the one-tailed Student’s *t*-test [38] with a 95% confidence interval.

## 3. Results

### 3.1. Simultaneous PGAA-Neutron Diffraction Results

#### 3.1.1. PGAA Results

**Figure 3:**
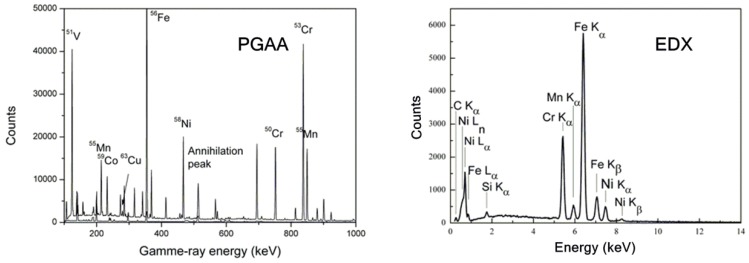
PGAA (**left**) and microscopy-energy dispersive X-ray spectroscopy (EDX) (**right**) spectra of G&H, with the identified peaks labelled with elemental symbols for EDX and isotopes for PGAA (measured with INES).

The background-subtracted spectrum of the G&H sample is presented in Figure 3 (right) with labels for identified elements/isotopes. Common features of standard PGAA spectra and their sources reveal the following: The peak at 511 keV is due to the electron-positron annihilation post pair-production [39]; the peak at 417 keV arises from Indium (In), commonly used in neutron spectroscopy to seal sample-holders, as well as inside the instrument’s HPGe detector [40], complemented by multiple Ge isotope peaks also from the HPGe detector. The major elements in stainless steels ^56^Fe-^50,53^Cr-^58^Ni, as well as the common alloying elements ^59^Co, ^55^Mn, ^63^Cu were also identified.

However, PGAA at a pulsed neutron source (e.g., ISIS) suffers from relatively large background contributions (Figure 3), which are intrinsic to the epithermal neutron beamlines and their traditional shielding set-ups [40,41]. Thus, comparative parallel PGAA measurements at BNC were carried out in order to quantify the elemental composition [28] (Table 1). From the bulk composition determined, both samples are majority-comprised of austenitic stainless steel (Fe-Cr-Ni), grade S30400/AISI 304, as per ASTM specification A240/A240M [42], also known as A2 or 18/8 stainless steel by alternative specifications. The content of the major elements Fe, Cr and Ni are not significantly different at *p* < .05, yet that of the alloying elements significantly differ at *p* < .05.

CF and PREN were calculated by applying the PGAA results to Equations (1) and (2), while the overall proportional errors were calculated using Equations (3) and (4), respectively. Mean and SD of CF and PREN together with their statistical analyses are reported in Table 2. As neither carbon nor nitrogen was detected using PGAA; their max values in ASTM specification A240/A240M [42] (Table 1) were used for these analytical determinations.

#### 3.1.2. Neutron Diffraction Results

**Figure 4:**
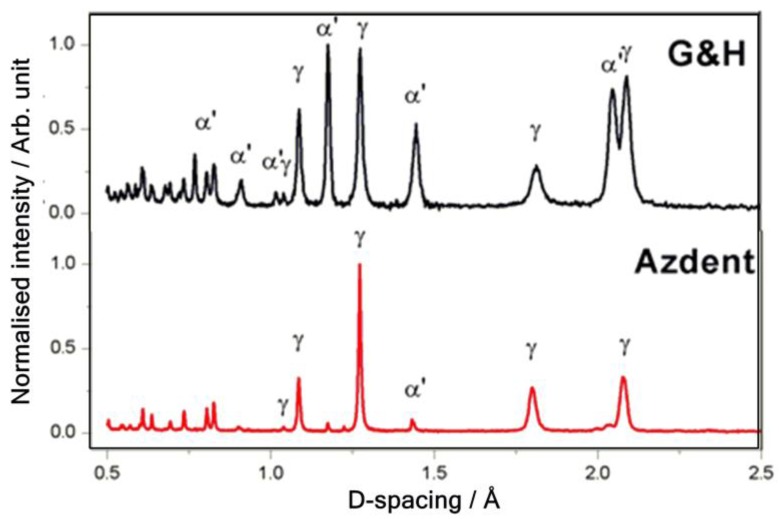
Diffraction patterns of G&H (**upper**) and Azdent (**lower**), with the main γ austenite and α’ martensite diffraction peaks labeled.

Diffractograms of the two samples are presented in Figure 4, with γ austenite and α’ martensite labelled at the proper peak positions. The quantified weight fractions and unit cell parameters previously reported [7] were used to calculate their respective individual volume fraction (vol%), tabulated in Table 2.

### 3.2. Simultaneous Elemental-Surface Morphology Measurement Results

#### 3.2.1. Surface Elemental Composition

The EDX spectrum of G&H is presented in Figure 3 (right), from which semi-quantitative elemental compositions were derived (Table 1). The content of the major elements Fe, Cr and Ni do not significantly differ at *p* < .05, but those of Mn and Si are significantly different at *p* < .05. Carbon (C) content is unrealistically high and should not be regarded on an absolute basis.

#### 3.2.2. Surface Morphology

**Figure 5:**
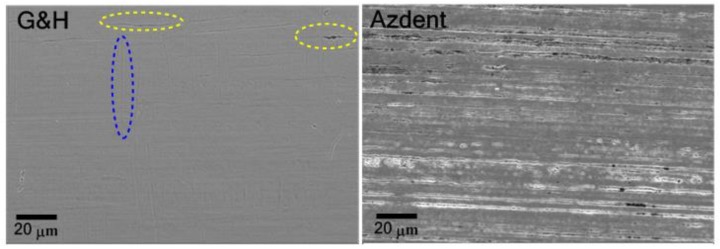
SEM micrographs of G&H (**left**) and Azdent (**right**) stainless steel archwires at 2000× magnification, scale bar 20 µm. Horizontal and vertical striations (along and perpendicular to the wire axis, respectively), as well as surface pitting are present in both samples, yet much less apparent or regular in G&H; dashed ovals assist in highlighting these features.

The SEM-determined surface morphology of the two samples are presented in Figure 5. The G&H surface appears featureless, yet after closer examination, shallow horizontal striations along the wire’s axis are apparent (Figure 5 (left side, top-right corner)), in addition to slight vertical striations. Sporadic pitting is also evident. Conversely, the Azdent surface is significantly more textured, with regular deeply roughened horizontal striations and regular pitting. Although surface morphology provides visual evidence of superficial defects and irregularities, the 2D nature of electron micrography interferes with quantitative characterization of the extent of irregularity and unevenness. Quantitative determination of the roughness was therefore conducted with AFM.

### 3.3. Surface Roughness Measurement Result

**Figure 6:**
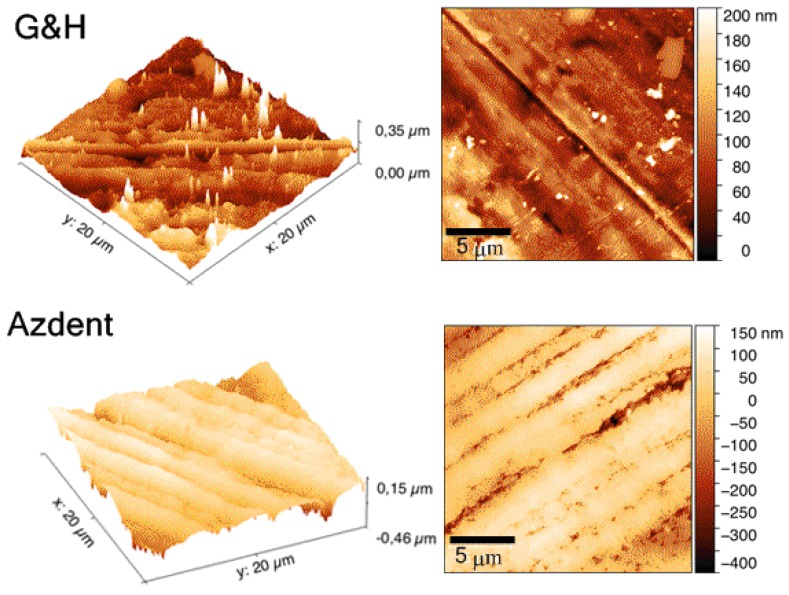
Atomic force microscopy (AFM) measurements of G&H (**upper**) and Azdent (**lower**) stainless steel archwires. Both 3D (**left**) and 2D (**right**) reconstructions of the surface morphology, (scale bar 5 µm), are shown. Azdent (lower) shows a much larger range deviation (Δ 0.61 μm vs. 0.35 μm for G&H) over matching domains (400 μm^2^), evidencing more pronounced grooving and pitting; in agreement with the SEM determinations.

The 3 D and 2 D reconstructions of the surfaces from AFM analysis are presented in Figure 6. It shows that both sample surfaces contain parallel striations, yet are deeper in Azdent, as evidenced by its larger range extent over an identical surface domain.

The surface roughness values and SD quantified with AFM are tabulated in Table 2. Azdent has higher surface roughness (RMS 60–82 nm, Ra 45–63 nm vs. RMS 30–38 nm, Ra 12–18 nm for G&H) (*p* < .05), in agreement with the trends revealed from the SEM morphology characterization (Section 3.2).

## 4. Discussion

### 4.1. Elemental Composition and Its Influence

PGAA is considered one of the most precise techniques for elemental composition analyses, whilst being advantaged by its non-destructive nature. Silicon, phosphorus, sulfur, nitrogen and carbon contents of the G&H and Azdent stainless steel archwire samples were all below the 1% detection limit of PGAA and thus not detected. Although complementary surface composition obtained with SEM-EDX detected carbon, it overestimated its content. SEM-EDX also detected silicon in both samples, within the 0.75% max [42]. PGAA detected a low content of Mo, Co and Cu in both samples, yet SEM-EDX did not. This is significant in that PGAA has higher detection sensitivity to alloying elements, facilitating their quantitative determination using Equations (1) and (2).

It is critical to note the EDX peak interference (Figure 3 (right), superimposed EDX peak at 0.5 keV) in contrast to the separated individual peaks with distinct element/isotope specific gamma energies (Figure 3 (left)). PGAA data analysis does not entail any peak deconvolution and thus can achieve higher precision than EDX can, which demands complicated deconvolution.

Further, the PREN value for G&H is significantly higher than Azdent (*p* < .05, Table 2), which may lead to higher resistance to pitting corrosion. Both techniques detected higher Cr and Ni contents in G&H (Table 1), which may lead to higher general corrosion resistance at room temperature.

Both samples contain relatively low alloy content compared to more highly alloyed types (e.g., AISI 310 (25Cr-20Ni)), thus the austenite is of the metastable type that exhibits a high degree of instability and is susceptible to rapid work hardening. In order to provide a quantitative measure of the austenite stability, we also calculated the CF from the PGAA results. The CF value for G&H was significantly lower than Azdent (*p* < .05, Table 2), evidencing its lower austenite stability and that the amount of martensite formed at room temperature, at true strain 0.2, was significantly higher than Azdent. Consequently, under the cold working conditions of wire production, G& H would be expected to demonstrate a greater work hardening deformation rate, and thus elevated strain-induced martensite formation.

### 4.2. Nanostructure and Its Influence

Structure-property (mechanical) relationships of metastable austenitic stainless steels are highly dependent on the extent of strain-induced martensitic transformation. Hence, precise determination of the martensite fraction is of utmost importance, yielding accurate predictions of the mechanical response of the bulk. Neutron diffraction offers the most accurate phase fraction determination of the sample bulk, due to deeper penetration depth (>10 mm in steels) compared with conventional XRD (<10 μm maximum in steel at 20 kV), making it greater than three orders of magnitude more comprehensive, providing bulk material analyses. Moreover, the sophisticated correction for preferred orientation necessary for quantitative determination using XRD is unduly simplified by GSAS [7].

The determined martensite fraction in the G&H sample was relatively high for a product marketed as an ‘austenitic stainless steel archwire,’ yet is comparable to the 56–82% volume range previously determined by XRD for other commercial products [11,21]. It can be concluded that commercial austenitic stainless steel archwire products commonly contain a fraction of martensite arising from work hardening. Its elevated martensite content predisposes it to higher stiffness, strength and hardness at the expense of limited ductility, formability and ultimately durability (toughness and fatigue life) in service under stress [43].

Based on the above observations, it is recommended that manufacturers track strain-induced martesite transformation during size reduction at room temperature, where cold working may induce rapid martensite transformation. This is particularly true for alloys with relatively low austenite stability, such as G&H. Intermediate thermal treatment helps restore ductility, allowing the material to undergo further cold working without cracking. For end product usage, low temperature heat treatment has been recommended to restore toughness, formability and resistance to fatigue, and also that sudden and excessive stretching and bending of wires is to be avoided [44].

Austenites are non-magnetic unless heavily cold-worked, which may result in slight magnetism; an additional characteristic that may be used as a probe for working history. G&H samples were found to be slightly magnetic, evidencing their having been heavily cold-worked.

### 4.3. Surface Characteristics and Its Influence

Roughness and surface irregularities have been evidenced as influencing both the biocompatibility and the performance of orthodontic archwires [45,46]. Specifically, that surface roughness and defects may be an indicator of the tendency towards corrosion [47,48], possibly contributing to archwire-bracket sliding friction, thus overall orthodontic component performance efficiency [49]. The SEM micrographs revealed that both samples have uneven surfaces with striations along the wire axis (horizontal, in Figure 5), ensuing from the drawing process. The Azdent surface had a higher irregularity density within the surface area captured in SEM and AFM measurements. The subsequent quantitative AFM-based roughness determinations (Table 2) confirmed that Azdent has a significantly rougher surface, in agreement with the SEM observations. This, combined with Azdent’s lower PREN value and lower general corrosion resistance at room temperature (due to lower chromium and nickel content), may lead to its inferior biocompatibility and corrosion resistance, as well as potential for inefficient sliding mechanics due to higher wire-bracket friction in orthodontic application.

### 4.4. Simultaneous Composition-Nanostructure Analyses with Neutron Spectroscopy

This work demonstrated the advantages of the simultaneous composition-nanostructure analysis (achieved with the INES beamline at the ISIS pulsed neutron and muon facility) as an efficient usage of allocated neutron experimental time, with limited radiation damage to samples. The high compositional resolution and trace element sensitivity simplified data analyses, whilst facilitating quantitative elucidation of the composition-structure-property relationship, through the calculation of CF and PREN, enabling performance prediction and a direct comparison between the two commercial products.

## 5. Conclusions

Through the novel combination of neutron scattering (diffraction and PGAA), SEM-EDX and AFM, we have evidenced a reproducible technique to access elemental composition, nano- through meso-scale structure, metallurgical structure, phase-composition and surface characteristics of two commercial stainless steel archwires used in dentistry/orthodontics and as medical materials. Taking advantage of PGAA’s higher compositional resolution and sensitivity to alloying-elements, we derived quantitative relationships: Composition–austenite stability, and composition–corrosion resistance. From those, it was elucidated that G&H’s lower austenite stability would lead to a higher work-hardening rate. Combined with the high martensite fraction, determined from neutron diffraction measurements (Section 3.1.2 and Figure 4), it may have been the result of extensive cold working and inadequate thermal treatment during material processing. The high martensite fraction would increase strength, yet at the expense of decreased ductility and formability, thus low temperature heat treatment is recommended to reduce the chances of premature failure. The insight gained herein may help equip practitioners with the understanding of the structure-property relationship, towards rational material selection and manipulation.

The approach and supporting methodology facilitated our work on relating fundamental physical principals with practical aspects of in-service material performance. The approach can be extended to the study of other metallic biomaterials used in medicine, dentistry and beyond, towards raising understanding of the impact of material processing on practical properties, providing performance predictions and rational guidance in appliance design.

## Figures and Tables

**Table 1:** Quantitative elemental composition as wt-% (SD) determined by neutron PGAA 238 and EDX, for the G&H and Azdent stainless steel archwire samples.

		Fe	Cr	Ni	Mn	Cu	Mo	Co	Si	C	N
**G&H**	PGAA	Bal. (0.60)	18.20 ^#^ (0.50)	9.60 (0.20)	1.28 (0.04)	0.39 (0.01)	0.18 (0.004)	0.033 (0.002)	0.75 ^a^	0.08 ^a^	0.1 ^a^
	EDX	Bal.	19.17	8.25	1.37	―	―	―	0.77	3.30	―
		(1.08)	(0.42)	(0.02)	(0.21)				(0.01)	(1.33)	
**Azdent**	PGAA	Bal. * (0.06)	17.60 * (0.50)	8.90 * (0.20)	2.50 ^.003^ (0.09)	1.10 ^.001^ (0.03)	0.03 ^.001^ (0.002)	0.28 ^.001^ (0.008)	0.75 ^a^	0.08 ^a^	0.1 ^a^
	EDX	Bal. *	19.04 *	7.79 *	2.56 ^.009^	―	―	―	0.41 ^.001^	3.28 *	―
		(0.42)	(0.01)	(0.17)	(0.06)				(0.02)	(0.57)	

^#^ PGAA results (apart from Mo) reproduced from [28], with permission from the Royal Society of Chemistry. ^a^ the values are taken from ASTM specification A240/A240M [42]; * denotes the differences are not statistically significant at *p* < .05; Superscripted values are *p* values and only the ones with significant differences at *p* < .05 are reported.

**Table 2:** The PGAA derived composition factor (CF) and pitting resistance equivalent number (PREN) with standard deviation (SD), phase composition and surface roughness parameters (SD) for the G&H and Azdent stainless steel archwire samples.

	Composition Effect		Phase Composition		Surface Roughness	
	**CF**	**PREN**	**γ Austenite**	**A’ Martensite**	**RMS (nm)**	**Ra (nm)**
**G&H**	17.37 (0.06)	18.79 (0.04)	53.80 vol%	46.20 vol%	33 (3)	14 (3)
**Azdent**	17.76 (0.09) ^.03^	17.70 (0.07) ^.003^	93.25 vol%	6.75 vol%	67 (8) ^.03^	50 (7) ^.02^

Superscripted values are *p* values for entries with significant differences at *p* < .05.
